# Quantitative Deficits of Preschool Children at Risk for Mathematical Learning Disability

**DOI:** 10.3389/fpsyg.2013.00195

**Published:** 2013-05-16

**Authors:** Felicia W. Chu, Kristy vanMarle, David C. Geary

**Affiliations:** ^1^Department of Psychological Sciences, University of MissouriColumbia, MO, USA; ^2^Interdisciplinary Neuroscience Program, University of MissouriColumbia, MO, USA

**Keywords:** approximate number system, quantitative knowledge, mathematics achievement, learning disability, dyscalculia, Title I preschool, executive control

## Abstract

The study tested the hypothesis that acuity of the potentially inherent approximate number system (ANS) contributes to risk of mathematical learning disability (MLD). Sixty-eight (35 boys) preschoolers at risk for school failure were assessed on a battery of quantitative tasks, and on intelligence, executive control, preliteracy skills, and parental education. Mathematics achievement scores at the end of 1 year of preschool indicated that 34 of these children were at high risk for MLD. Relative to the 34 typically achieving children, the at risk children were less accurate on the ANS task, and a one standard deviation deficit on this task resulted in a 2.4-fold increase in the odds of MLD status. The at risk children also had a poor understanding of ordinal relations, and had slower learning of Arabic numerals, number words, and their cardinal values. Poor performance on these tasks resulted in 3.6- to 4.5-fold increases in the odds of MLD status. The results provide some support for the ANS hypothesis but also suggest these deficits are not the primary source of poor mathematics learning.

## Introduction

The poor mathematical skills of nearly one in four adults in many modern economies places them at heightened risk for underemployment and frequent unemployment, controlling for reading ability, intelligence, and race (Rivera-Batiz, 1992; Bynner, 1997). These employment-related risks can, in many cases, be traced to poor numerical competencies at school entry (Duncan et al., 2007; Morgan et al., 2009); children who start school with a poor understanding of numerals are four times more likely than their peers to score in the bottom quartile on employment-relevant quantitative tests by adolescence, controlling for other factors (Geary et al., 2013). Consequently, reducing this long-term risk may require identification and eventually the remediation of prekindergarten precursors of these school-entry deficits.

One hypothesis is that poor school-entry number knowledge results from deficits in systems for representing the approximate quantity of collections of items [approximate number system (ANS); Gilmore et al., 2010; Piazza et al., 2010; Mazzocco et al., 2011b] or the ability to quickly apprehend the exact quantity of small sets of items (subitizing; Koontz and Berch, 1996; Landerl et al., 2003; Butterworth, 2005; Iuculano et al., 2008). It is currently debated whether subitizing is dependent on the ANS or is a distinct system, and for ease of presentation we assume a single system. The ANS is thought to underlie humans’ intuitive sense of numerical magnitude (Gallistel and Gelman, 1992, 2000; Feigenson et al., 2004). We tested the hypothesis that a deficit in this system contributes to poor mathematics achievement by comparing preschool children at risk for a mathematical learning disability (MLD) to a group of their typically achieving (TA) peers on a measure of ANS acuity. As a contrast to any ANS deficit, the groups were also compared on other quantitative competencies that have been shown to be predictive of later mathematics achievement.

The acuity of the ANS is assessed by children’s sensitivity to subtle differences in the relative magnitudes of collections of objects, and individual differences in this sensitivity may be correlated with mathematics achievement. For example, ninth graders’ ANS acuity was found to be retrospectively correlated with standardized mathematics achievement scores as far back as kindergarten, controlling for intelligence, executive functions, and other factors (Halberda et al., 2008). A follow-up study revealed that ANS acuity was particularly poor for adolescents with MLD, again controlling for working memory and other factors (Mazzocco et al., 2011a). In addition, some studies have found that school-age children with MLD may have deficits or delays in the ANS system (Landerl et al., 2003; Mazzocco et al., 2011b). Piazza et al. (2010) found that ANS sensitivity of 10-year-olds with MLD was about the same as that of TA 5-year-olds matched on intelligence.

Other studies, however, have found no evidence of an ANS deficit in children with poor mathematics achievement generally or MLD in particular (Rousselle and Noël, 2007; Iuculano et al., 2008). These studies and related ones suggest that individual differences in mathematics achievement and MLD may instead be due to deficits or delays in the explicit attentional sensitivity to or understanding of Arabic numerals, number words, and the relations among them (Hannula et al., 2010; Bugden and Ansari, 2011; De Smedt and Gilmore, 2011; Lyons and Beilock, 2011). The extent to which ANS sensitivity contributes to the initial learning of this explicit quantitative knowledge remains to be determined (Gilmore et al., 2010; Geary, 2013), but at the very least these studies indicate that the testing of the ANS hypothesis needs to be done in the context of a broad assessment of basic quantitative knowledge and skills. In this way, the relative contribution of ANS sensitivity to poor mathematics achievement can be contrasted with the relative contribution of other quantitative competencies.

Candidates for these other competencies include those that have been identified in studies of infants and preschool children (e.g., Gelman and Gallistel, 1978; Strauss and Curtis, 1984; Wynn, 1990; vanMarle, 2013), especially those with a demonstrated link to later mathematics achievement (Locuniak and Jordan, 2008; Jordan et al., 2009b; Krajewski and Schneider, 2009; LeFevre et al., 2010). For instance, using quantitative tasks that assess children’s skills at counting objects, knowledge of counting principles, numeral recognition, and simple non-verbal addition and subtraction, Jordan and colleagues found that early mathematical knowledge and growth in this knowledge was predictive of mathematics achievement in second and third grade (Locuniak and Jordan, 2008; Jordan et al., 2009b).

The preschool predictors of risk for later MLD are not currently known, but children scoring in the bottom 25% on mathematics achievement tests and especially those scoring in the bottom 10% are likely at risk (Geary et al., 2007; Murphy et al., 2007). These cutoffs are based on studies of school age children and adolescents who have difficulties learning mathematics. Students with MLD include as many as 7% of children and adolescents (ranging from 4 to 14% depending on classification methods), and consistently (across grades) score at or below the 10th-percentile on mathematics achievement tests (Lewis et al., 1994; Barbaresi et al., 2005; Shalev et al., 2005). An additional 10% or so of children are persistently low achieving (LA) and score between the 11th and 25th percentiles in mathematics across grades, despite average intelligence and reading ability (for reviews, see Dowker, 2005a; Berch and Mazzocco, 2007).

In a 5-year prospective study, Geary et al. (2012b) found that children with MLD and their LA peers represented different cut points on the normal distribution of mathematical achievement. For some numerical or arithmetical competencies the children in these groups showed similar deficits, relative to TA children, whereas in others the LA children showed more rapid across-grade gains than the children with MLD. Other studies have also found different numerical and arithmetical patterns of strengths and deficits within MLD and LA groups, and even within TA groups (Denvir and Brown, 1986; Dowker, 2005b; Jordan et al., 2009a; Geary et al., 2012a). The results suggest that MLD and LA may represent different levels of severity for some basic numerical deficits, different patterns of deficit for others, and that even within such groups there are often quantitative strengths as well as deficits.

The present study focused on the quantitative development of children at the beginning and the end of their first year of Title I preschool. Title I is a federally (United States) funded program that provides services to children at risk for school failure, and thus includes a disproportionate number of children who are likely at risk for later MLD or LA (hereafter, MLD). In addition to a broad assessment of quantitative competencies, including ANS sensitivity, we also assessed other factors that have been shown to influence mathematical learning; specifically, intelligence (Deary et al., 2007; Geary, 2011) and executive functioning (Blair and Razza, 2007; Bull et al., 2008; Clark et al., 2010). These were used as covariates in our contrasts of MLD and TA groups, along with measures of preliteracy skills and parental education.

## Materials and Methods

### Participants

Seventy-one children were recruited from the Title I preschool program within the public school system in Columbia, MO, USA; data for two children were excluded due to very low (<61) intelligence scores and one other moved. The results presented are based on the remaining 68 (35 boys) children. Title I Preschool is a federally funded program offering services to 3- to 5-year-old children with developmental needs, and is designed to prepare them for successful school entry. The Columbia Public Schools Title I Preschool program serves about 750 children, with 26 classrooms located throughout the district. Consent forms were sent to all entering 3-year-olds (∼240 children), and the sample consisted of those whose parents consented to participation. At the time of the first assessment, the children were 3 years 9 months of age (range: 3y2m–4y2m).

Demographic information was obtained through parent survey for a subset (*n* = 51) of the sample. Of those parents who returned the survey, not all provided responses to all questions and thus the number of responses varied by question. The ethnic composition of the sample was 81% non-Hispanic, 10% Hispanic/Latino, and the remaining unknown. The racial composition was 60% White, 20% Black, 8% Asian, 8% more than one race, and 4% unknown. The self-reported total household income was: $0–$25k (35%), $25k–$50k (21%), $50k–$75k (25%), $75k–100k (15%), $100k–$150k (2%), $150k or more (2%). Thirty-three percent of respondents reported receiving food stamps, and 8% reported receiving housing assistance.

From the survey, we were most interested in parental education (*n* = 49). The highest level of mothers’ education was: some high school (10%), complete HS/GED (48%), bachelor’s degree (30%), post-graduate degree (12%). The highest level of fathers’ education was some high school (12%), complete HS/GED (43%), bachelor’s degree (18%), post-graduate degree (27%). Maternal and paternal education levels were found to be highly correlated, *r*_48_ = 0.80, *p* < 0.0001, and thus we created a mean parental education variable (α = 0.88). Three groups were then created from this variable: no information provided (*n* = 19), high school (*n* = 25), and college (*n* = 24). The high school group consisted of children who had at least one parent with a high school diploma or equivalent but no better, and the college group consisted of those with at least one parent who was a college graduate (or better).

### Materials

#### Quantitative tasks

Our quantitative tasks were administered in two sessions, each conducted once in Fall and once in Spring, for a total of four sessions. These sessions assessed verbal and non-verbal counting, numeral recognition, ordinality, cardinality, magnitude sensitivity, and informal arithmetic.

##### Counting

We assessed children’s conceptual knowledge and procedural skills using three tasks: enumeration, verbal counting, and counting knowledge. For the *enumeration* task, children were shown an array of 20 stickers and asked to count them, pointing to each one. The score was the highest number counted before committing an error. The *verbal counting* task involved the child reciting the count list, starting from “one” and counting as high as they could without an error, or until they reached 100. This task determined how well the child had the count list committed to memory. The *counting knowledge* task assessed children’s understanding of basic counting concepts (e.g., one-one correspondence; Gelman and Gallistel, 1978) and their awareness of essential and unessential features of counting (Briars and Siegler, 1984). On each of 13 trials, children watched a puppet count a line of checker pieces (alternating in color, red and black) and then indicated whether the count was “OK,” or “Not OK and wrong.” There were four types of trials: correct (four trials), right-left (four trials), pseudo-error (five trials), and error (four trials) (Geary et al., 1992). For correct trials, checkers were counted sequentially and correctly, from left to right. In right-left trials, checkers were counted sequentially and correctly, from right to left. Pseudo-error trials consisted of counting the pieces correctly from left to right, starting first with one color and then returning to the left side of the array and continuing with the other color. For error trials, checkers were counted sequentially from left to right, but the first checker was counted twice. The score was the overall percent of trials correctly identified as “OK” (i.e., correct, right-left, pseudo-error) or “not OK and wrong” (i.e., error).

##### Numeral recognition

For the *numeral recognition* task, children were shown the Arabic numerals (one-at-a-time) from 1 to 15 in random order. Children were asked to name each one, and the score was the total number of numerals correctly named. Only the numerals correctly identified were used in the *numeral comparison* task (below).

##### Ordinality

Two tasks were used to assess ordinality. The *numeral comparison* task targeted children’s understanding of ordinality by asking them to compare two Arabic numerals and report “which is bigger?” Each child completed six comparisons. The score was the total percent correct across the six trials. This task tested whether children have mapped Arabic numerals onto non-verbal quantities and whether they understand the numerals as an ordered sequence.

The second task was the *ordinal choice* task. This task was based on a common procedure that has been used successfully with preverbal infants with small (Feigenson et al., 2002) and large set sizes (vanMarle and Wynn, 2011; vanMarle, 2013) and with non-human primates (vanMarle et al., 2006). Children watched an experimenter sequentially hide two different numbers of objects (e.g., small toy fish) in two opaque cups; items were dropped into the cups one at a time. The children were then asked to pick the cup that contained more objects. There were six different comparisons (1 vs. 2, 2 vs. 3, 3 vs. 4, 4 vs. 5, 5 vs. 6, and 6 vs. 7). In order to successfully identify the larger quantity, children had to mentally track the sum and compare the number of objects in each cup. Because the comparisons varied in difficulty (i.e., ratios varied from 0.5 to 0.86), we generated a single score that was weighted for the difficulty of the comparison. This was done by first multiplying each trial’s score (incorrect = 0, correct = 1) by the ratio of the comparison (e.g., 2 vs. 3 = 0.67) and then summing the products across trials.

##### Cardinality

Children completed two tasks that assessed their knowledge of cardinal value (Wynn, 1990; Sarnecka and Carey, 2008). In the *give-a-number* task (Wynn, 1990), children were asked to give the experimenter exactly 1, 2, 3, 4, 5, and 6 objects from a pile. Children began at set size 1 and advanced to the next set size after a correct response; if they were incorrect, they went down one set size. The highest number of objects they correctly gave the experimenter on at least two of three attempts was taken as the highest set size for which the child understood cardinality (Le Corre and Carey, 2007).

The second task, *point-to-x* (Wynn, 1990), required children to “point to the picture that has *x* objects.” Children received two blocks of six trials with ratios ranging from 0.5 to 0.67 (1 vs. 2, 5 vs. 10, 2 vs. 3, 6 vs. 9, 4 vs. 7, and 5 vs. 8), with both exclusively large and small sets represented. On each trial, children saw two sets of pictured objects on a laptop display (one on the left and one on the right). The smaller number was the target on half of the trials, and the side on which the smaller set was displayed was counterbalanced across trials. The score was determined by multiplying each trial’s score (incorrect = 0 or correct = 1) by the ratio of the comparison (e.g., 5 vs. 10 = 0.5). Products were summed across trials to produce a single score weighted for the difficulty of the comparison.

##### Magnitude sensitivity

Magnitude sensitivity was tested using a discrete quantity discrimination task (hereafter, ANS task) and a continuous quantity discrimination task. The ANS task assessed the precision with which children mentally represent discrete quantities of objects. Using the Panamath program (Halberda et al., 2008), children received 24 test trials on a laptop computer. Each trial contained two sets of blue and yellow dots (each set was contained within a rectangle), and children identified which set “had more dots.” All dot displays consisted of more than three dots and were displayed for only 2533 ms in order to discourage verbal counting. Ratios of blue:yellow dots were randomly selected for each trial and varied between 1.29 and 3.38. The Panamath program provides estimates of children’s Weber fraction (*w*), which is thought to index ANS acuity or the precision with which one can represent a given quantity, and percent correct (see Halberda and Feigenson, 2008).

The continuous quantity discrimination task was conceptually similar, but children were asked to discriminate a continuous quantity, surface area. Children were presented with 24 test trials; in each trial, they were presented with a rectangle made of blue and red squares (four trials at each of six red:blue ratios – 1:4, 1:3, 1:2, 2:3, 3:4, and 4:5). For each trial, children reported whether there was “more red” or “more blue” in the picture.

##### Informal arithmetic

Children’s early arithmetic skills were assessed using two tasks. The *magic box* task (vanMarle and Wynn, 2001) is a variant of Starkey’s (1992) search-box task and was designed to assess children’s implicit understanding of addition and subtraction. Children were first introduced to a puppet that they were told would sometimes perform a magic trick on items hidden in a box. Unbeknownst to the child, a false floor inside the box could be manipulated to create the illusion that an object had appeared or disappeared when the lid was closed. On each trial, the child watched an experimenter hide 0, 1, or 2 objects in the box. The experimenter then added or removed an object from the hidden set as the child watched. Children were not allowed to see the resulting set. The lid was closed and then opened to reveal either the correct result of the operation, or the incorrect result. There were eight trials in this task, with a correct and an incorrect result for each of four problems: 0 + 1 = 1 or 0, 1 + 1 = 2 or 1, 1−1 = 0 or 1, 2−1 = 1 or 2. When the result was revealed, children were asked whether the puppet had done a magic trick. In order to correctly identify the incorrect outcomes as magical and the correct outcomes as not magical, the child needed to understand the effects of addition or subtraction of an object on set size. This task only required children to detect whether the operation was correct or incorrect, and did not require them to predict the exact result of the operation.

The second task was *non-verbal calculation* (Levine et al., 1992). Here, children were shown addition or subtraction of one or more disks from a hidden set of disks and then asked to predict the exact numerical result. Children watched an experimenter place a number of plastic disks in a line; the experimenter then covered the disks with a plate and added or removed some from under the plate. Children were asked to create a set of disks equal in number to the hidden set, but could also report the answer verbally. After four familiarization trials in which the children simply matched a hidden set, there were 12 test trials, presented in random order: 3−1, 2 + 2, 4−2, 1 + 3, 4−1, 4 + 1, 3 + 2, 1 + 4, 5−2, 5−3, 2 + 4, and 6−4.

#### Cognitive measures

Children completed a cognitive battery to control for intelligence, executive control, and preliteracy skills.

##### Intelligence

The children were administered the Receptive Vocabulary, Block Design, and Information subscales of the *Wechsler Preschool and Primary Scale of Intelligence – III* (WPPSI; Wechsler, 2002). Following standard procedures, scores were scaled and prorated to generate an estimate of Full Scale IQ (intelligence).

##### Executive control

Executive function was assessed using the Conflict EF scale developed for children from 2 to 6 years of age (Beck et al., 2011). This scale consists of six levels; the first four included two subsections (five trials each), whereas Levels 5, 6A, and 6B included 10 trials each. All children began on Level 2 following age-based procedures.

The Conflict EF scale consisted of a card-sorting task. Children were presented with two black plastic index card boxes with holes cut into the top; each box had a target card affixed to the front. Children were given a rule and asked to place a card in the appropriate box. Each level consisted of normal sorting trials, followed by conflict trials. For example, children placed the card in the corresponding box depending on whether the card was a “big kitty” or a “little kitty”; in conflict trials, children were asked to switch the rule, i.e., a “big kitty” would go in the “little kitty” box. In subsequent levels, children sorted the cards depending on shape or color of the card (again, the rule was reversed to create a conflict trials). More advanced levels required children to sort cards according to shape or color depending on whether a black border was present or absent on the card. In order to move on to the next level, children had to complete four out of five trials correctly; in levels with 10 trials, children had to complete four shape trials and four color trials correctly in order to proceed to the next level. The score was the total number of correct conflict trials.

##### Preliteracy

To assess children’s preliteracy skills, one subtest of the *Phonological Awareness Literacy Screening-PreK* (PALS; Invernizzi et al., 2004), Upper-Case Alphabet Recognition, was administered. This task was chosen because it is a reliable indicator of later reading ability (Blatchford et al., 1987). Children were presented with capital letters in the alphabet (a few at a time) and asked to identify each letter. The score was the total number of letters correctly identified.

#### Mathematics achievement groups

In order to identify mathematics achievement groups, participants also completed the *Test of Early Mathematical Ability-3* (TEMA-3; Ginsburg and Baroody, 2003), which is a nationally normed (*M* = 100, SD = 15) measure of young children’s mathematical competencies. Items on the TEMA-3 included producing finger displays to represent different quantities, counting, and making numerical comparisons. All children started on the first item of the test and continued until they failed five consecutive items.

There was a break in the distribution of TEMA scores between the 21st and 27th national percentile ranks; thus, children with scores less than the 22nd percentile were categorized as at risk for MLD (*n* = 34) and the remaining children categorized as TA (*n* = 34). The respective mean percentile ranks on the TEMA were 9th and 56th for the MLD and TA groups [*F*(1, 66) = 146.09, *p* < 0.0001], consistent with ranks found for older children with MLD (Geary et al., 2012b). The groups differed in intelligence (*p* < 0.0001), executive functions (*p* < 0.05), and letter identification scores (*p* < 0.0001), as shown in Table 1; however, the difference on the TEMA remained significant when these scores were covaried (lsmeans = 13th and 51st respective percentile rank, *p* < 0.0001). In contrast, there were no group differences in level of parental education, χ^2^(6) = 8.25, *p* = 0.2207.

**Table 1:** **Academic and ability scores across MLD status**.

Test	Group	
	MLD	TA
Intelligence	92 (13)	104 (17)
TEMA	79 (6)	102 (9)
Executive functions	28 (12)	34 (14)
Letter identification	6 (7)	17 (9)
Parental education	3.5 (1.0)	3.6 (0.8)

*Parenthetical values are standard deviations. MLD, mathematical learning disability; TA, typically achieving; TEMA, standard scores (*M* = 100, SD = 15) from the *Test of Early Mathematical Abilities-3* (Ginsburg and Baroody, 2003); Executive functions scores are the mean number of correct conflict items with a minimum possible score of 15 and a maximum of 60*.

Because the TEMA-3 and our quantitative tasks are based on the same research literature, there is some overlap in the assessed competencies. For the ages assessed here, our tasks cover a broader range of competencies and include more difficult items for overlapping ones. The mean and standard deviation of the raw score of the MLD group indicated that they were, on average, successful on TEMA-3 items that involved identifying a set of up to three items, showing the examiner up to five fingers, counting to five, and identifying *more* when comparing simultaneously presented sets less than 11. The primary overlap is for our enumeration and verbal counting tasks, and in both cases our range of potential counts is higher than the items on the TEMA-3. Conceptually the *more* task overlaps with our ordinality tasks. However, there are no explicit numeral comparison items at this point in the TEMA-3, and our ordinal choice task involves comparisons of sets of items with sequential presentation and smaller (i.e., more difficult) ratios between sets than the TEMA-3 *more* item.

The mean and standard deviation of the TEMA-3 score of the TA group indicated that they were, on average, able to answer additional items that assessed counting up to 10, cardinal knowledge using counting and give-a-number, numeral identification, and non-verbal calculation. The three latter items are similar to our tasks, but our tasks included more items and somewhat more difficult items. For the ages assessed here, most of the children would not have been administered TEMA-3 items that overlapped with the majority of our tasks, including counting knowledge, numeral comparison, point-to-x, discrete (ANS task) and continuous magnitude, or magic box tasks.

### Procedure

Children were tested individually in six testing sessions lasting approximately 35 min each. All sessions were completed in their preschool facility. *Quant 1* [enumeration, give-a-number, point-to-x, magic box, discrete quantity discrimination (ANS), and ordinal choice, in that order] and *Quant 2* (verbal counting, non-verbal calculation, numeral recognition, numeral comparison, counting knowledge, and continuous quantity discrimination, in that order) were each administered, in separate sessions, once at the beginning of the fall semester and once in the middle of the spring semester. At the beginning of the spring semester, children were tested in a single session that included the EF scale (Beck et al., 2011), the WPPSI-III (Wechsler, 2002), and letter identification (Invernizzi et al., 2004). The final testing session consisted of the TEMA-3 (Ginsburg and Baroody, 2003), and was administered at the end of the spring semester. The sequence of testing and mean ages at each assessment are provided in Table 2. The experimental procedure was reviewed and approved by the Institutional Review Board of the University of Missouri. Written consent was obtained from all parents, and all participants provided verbal assent for all assessments.

**Table 2:** **Sequence of tasks and ages**.

Sequence of tasks	Age of children
Quant 1 (fall)
Enumeration	Mean: 3y9m
Give-a-number	Range: 3y2m–4y2m
Point-to-x	
Magic box	
Discrete quantity discrimination (ANS)	
Ordinal choice	
Quant 2 (fall)
Verbal counting	Mean: 3y11m
Non-verbal calculation	Range: 3y4m–4y3m
Numeral recognition	
Numeral comparison	
Counting knowledge	
Continuous quantity discrimination	
Cognitive battery
Executive functions (card-sorting)	Mean: 4y0m
WPPSI-III (receptive vocabulary, block design, information)	Range: 3y6m–4y5m
PALS (upper-case alphabet recognition)	
Quant 1 (spring)	Mean: 4y2m
	Range: 3y6m–4y7m
Quant 2 (spring)	Mean: 4y2m
	Range: 3y7m–4y8m
*Test of early mathematics ability-3*	Mean: 4y3m
	Range: 3y8m–4y8m

In order to encourage children and keep them motivated during testing sessions, they received stickers after completion of tasks or blocks of trials; at the end of testing sessions, children also received educational prizes (e.g., age-appropriate books). All sessions were videotaped and the video records were used for coding and to determine reliability. Trained observers naïve to the purpose of the study reviewed testing sessions and recorded data for 15 randomly selected participants. Reliability was calculated separately for each of the 12 quantitative tasks by correlating the data collected during the test session with that recoded from the videotapes. Reliability was >0.92 for all the tasks, with one exception (time 2 Counting Knowledge = 0.86). Given the very high reliabilities, all analyses were conducted with the data collected during the testing sessions.

### Analyses

Missing observations (6%) were estimated (maximum likelihood estimates with five imputations) using the multiple imputations program of SAS Institute (2004). To reduce the number of quantitative variables and the risk of false positives, the 12 time 1 and time 2 quantitative tasks were submitted to a principal components factor analysis (promax rotation) and factors with Eigenvalues >1 were retained (Gorsuch, 1983). The enumeration, give-a-number, numeral recognition, and verbal counting tasks loaded on the same factor at both times of measurement, and thus composite number knowledge factor scores were created using the mean of these four tasks for time 1 (α = 0.80) and time 2 (α = 0.85). In addition, overall scores for the TEMA were regressed on the time 1 number knowledge composite along with the eight remaining individual quantitative task variables using a stepwise procedure (forward selection with *p* < 0.05 to enter and stay). We focused on time 1 because competence at the beginning of preschool is of greater practical and theoretical importance than that assessed toward the end of a year of preschool (Libertus et al., 2011). The number knowledge composite (*r^2^* = 0.57, *p* < 0.0001) was selected first, followed by ordinal choice (*pr^2^* = 0.06, *p* < 0.002); no other quantitative variables were selected. Thus, all subsequent analyses included the number knowledge composite and ordinal choice variables, along with the Weber fraction and percent correct variables from the ANS task. The latter were included based on our *a priori* prediction of poor ANS acuity for children with MLD.

Following analyses of group differences (MLD vs. TA) on the ANS task Weber fraction and percent correct variables, random intercept mixed models were run to estimate group differences on the number knowledge composite and ordinal choice variables across time 1 and time 2, using age, sex, parental education, intelligence, executive control, and letter identification scores as covariates. Logistic regressions were then used to determine the odds of MLD status using the time 1 number knowledge composite and ordinal choice variables along with ANS percent correct as predictors, using the same covariates as in the mixed models. The intelligence, executive control, and letter identification variables were standardized (*M* = 0, SD = 1) for all of the analyses. Regressing mathematics achievement on the Weber fraction and percent correct variables resulted in a significant relation for percent correct (*p* = 0.0091) but not the Weber variable (*p* = 0.8584). Thus, only the percent correct variable was used in the logistic regressions.

## Results

The first set of analyses addresses the hypothesis that children at risk for MLD have deficits in the ANS system, whereas the second set addresses group differences in rate of development of the quantitative competencies assessed by the number knowledge composite and the ordinal choice variables. In the third set, we focus on the predictive utility of the ANS percent correct, number knowledge composite, and ordinal choice variables for predicting the odds of MLD status at the end of 1 year of preschool. In the final section, we present an assessment of variability in task performance for children in the MLD group to determine if quantitative deficits are uniform or variable for these children.

### Mathematical learning disability and ANS acuity

Median Weber fractions (*w* = 0.71, 0.59, for time 1 and time 2, respectively) and percent correct (63, 66% correct for time 1 and time 2, respectively) were consistent with previous studies of children of the same age (Libertus et al., 2011; Mazzocco et al., 2011b). However, many children had difficulty with the task and thus the Weber fractions are based on only 48 and 45 of the 68 children for time 1 and time 2, respectively. To increase the amount of useable data, we took each child’s best (smallest) Weber fraction and highest percent correct across the two times of measurement, resulting in a median Weber fraction of 0.56 (*n* = 61) with 75% correct (*n* = 68).

Children at risk for MLD had higher mean Weber fractions (*M* = 1.65, SD = 1.61) than their TA peers (*M* = 0.84, SD = 1.06), *F*(1, 66) = 6.05, *p* < 0.02, *d* = 0.61, and they were less accurate on the ANS task (*M* = 69%, SD = 14) than the TA children (*M* = 81%, SD = 16), *F*(1, 66) = 10.67, *p* < 0.002, *d* = 0.79. Control of group differences in intelligence, executive functions, and letter identification scores resulted in a non-significant group difference for the Weber fraction (*p* = 0.2019), but the difference in percent correct remained significant (*p* = 0.029, lsmeans = 71 and 80% for the MLD and TA groups, respectively).

As a follow-up, we created subgroups of MLD (*n* = 16) and TA (*n* = 12) children with intelligence scores between 90 and 110. These subgroups did not differ on intelligence (*M* = 98, SD = 5; *M* = 100, SD = 6 for the MLD and TA groups respectively) or executive functions (M = 27, SD = 12; *M* = 33, SD = 13) (*p*s < 0.2303), but they did for letter identification scores (*M* = 9, SD = 8; *M* = 17, SD = 7, *p* < 0.0285). The mean standard mathematics achievement score was 81 (SD = 5.2) for the MLD subgroup and 103 (SD = 11) for the TA subgroup (*p* < 0.0001). The MLD subgroup had a higher mean Weber fraction (*M* = 1.35, SD = 1.57) than the TA subgroup (*M* = 0.58, SD = 0.75) but the difference was not significant (*p* = 0.129). Mean percent correct was 74% (SD = 15) for the MLD subgroup and 87% (SD = 13) for the TA subgroup, which was significant (*p* = 0.0227, *d* = 0.93).

### Mathematical learning disability and quantitative development

**Figure 1:**
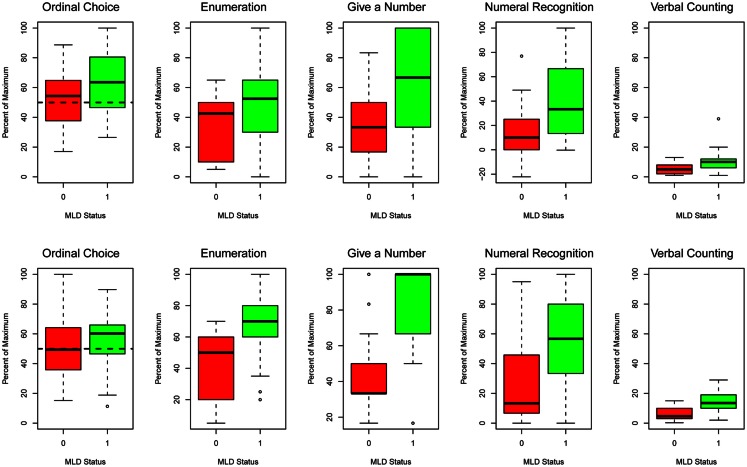
**Boxplots for the scores of the MLD (0) and TA (1) groups for the ordinal choice (the dashed line indicates 50% chance performance) and four quantitative tasks (Enumeration, Give a number, Numeral Recognition, and Verbal Counting) that compose the number knowledge composite**. Time 1 is on top and time 2 on the bottom.

Mean scores (percent of maximum scores) across times of measurement for the MLD and TA groups for the ordinal choice and four quantitative tasks that compose the number knowledge composite are shown in Figure 1. The second and third columns of Table 3 show the summary results for group differences on the number knowledge composite. Controlling for other variables in the model, number knowledge composite accuracy was 13.8% lower at time 1 than time 2 (*p* < 0.0001) and the children at risk for MLD scored 14.1% lower than their TA peers at time 1 (*p* < 0.0001). The gap between the MLD and TA groups widened to 20% (14.1 + 5.9) by time 2 (*p* < 0.0050). Follow-up analyses of the four individual tasks revealed the MLD/TA contrast was significant for enumeration (*p* < 0.0129), give-a-number (*p* < 0.0001), and verbal counting (*p* < 0.0001), as was the interaction between time and MLD contrasts for enumeration (*p* < 0.0155), give-a-number (*p* < 0.0296), and verbal counting (*p* < 0.0312). For all of the latter tasks, the gap between the MLD and TA groups widened from time 1 to time 2.

**Table 3:** **Mixed model results for the number knowledge composite and ordinal choice tasks**.

Variable	Number knowledge composite		Ordinal choice	
	Estimate	*p*	Estimate	*p*
Intercept	17.0 (20.2)	0.4036	46.5 (32.3)	0.1549
Time 1 vs. time 2	−13.8 (1.47)	0.0001	5.99 (3.37)	0.0782
Age	0.71 (0.44)	0.1124	0.34 (0.71)	0.6318
Girl vs. boy	−1.35 (2.46)	0.5844	1.62 (3.93)	0.6804
NI vs. college	0.34 (3.29)	0.9180	−3.01 (5.25)	0.5681
HS vs. college	1.72 (3.06)	0.5751	−5.74 (4.89)	0.2424
Intelligence	5.10 (1.67)	0.0028	−0.38 (2.67)	0.8869
Executive functions	0.56 (1.49)	0.7067	2.22 (2.39)	0.3534
Letter identification	7.01 (1.56)	0.0001	−5.72 (2.50)	0.0235
MLD vs. TA	−14.1 (2.93)	0.0001	−9.64 (4.83)	0.0478
MLD by time	5.94 (2.08)	0.0050	−6.43 (4.77)	0.1797

*Parenthetical values are standard errors. NI, no information on parental education; HS, high school; MLD, mathematical learning disability; TA, typically achieving. Negative values indicate the contrasted group (e.g., girls) had lower scores than the contrast group (e.g., boys), and positive values indicate the opposite*.

The two rightmost columns of Table 3 show that the children at risk for MLD scored 9.6% lower than their TA peers on the ordinal choice task at time 1 (*p* = 0.0478), with no significant change in this deficit across time 1 and time 2. Follow-up analyses indicated the children at risk for MLD did not score above chance (50%) for either time 1 (*t*_33_ = 0.57, *p* = 0.5744) or time 2 (*t*_33_ = 0.64, *p* = 0.5241) on the ordinal choice task, but the TA children did; time 1 (*t*_33_ = 3.75, *p* = 0.0007), time 2 (*t*_33_ = 2.22, *p* = 0.0331).

### Quantitative performance and odds of MLD status

Approximate number system task percent correct, and time 1 ordinal choice and number knowledge composite scores were used in separate analyses to predict the odds of MLD status, controlling for sex, parental education, intelligence, executive functions, and letter identification scores. A 1 SD decrease in ANS task percent correct was associated with a 2.4-fold increase in the odds of being classified as at risk for MLD [χ^2^(1) = 5.42, *p* = 0.0199, 95% CI, 1.15–5.12]. The corresponding estimates for the ordinal choice and number knowledge composite variables were 3.6 [χ^2^(1) = 6.77, *p* = 0.0093, 95% CI, 1.37–9.55] and 4.5 [χ^2^(1) = 6.04, *p* = 0.014, 95% CI, 1.36–15.11], respectively. Simultaneously estimating all three quantitative variables produced a significant likelihood ratio for the overall model, χ^2^(9) = 45.2, *p* < 0.0001, and the estimates for the number knowledge composite and ordinal choice variables were significant, as shown in Table 4.

**Table 4:** **Estimates from logistic regression**.

Variable	Estimate	*p*	Odds ratio	95% CI
Intercept	−3.16 (1.38)	0.0219	–	–
Girl vs. boy	0.49 (0.41)	0.2292	2.7	0.53–13.29
NI vs. college	−0.19 (0.57)	0.7376	2.4	0.32–17.03
HS vs. college	1.24 (0.67)	0.0637	9.8	0.99–97.89
Intelligence	−0.16 (0.50)	0.7539	0.9	0.32–2.29
Executive functions	−0.71 (0.48)	0.1386	0.5	0.19–1.26
Letter identification	1.54 (0.56)	0.0056	4.7	1.57–13.96
ANS percent correct	0.28 (0.49)	0.5681	1.3	0.51–3.43
Ordinal choice	1.40 (0.55)	0.0111	4.1	1.38–12.00
Number composite	1.96 (0.83)	0.0189	7.1	1.38–36.46

*Parenthetical values are standard errors. NI, no information on parental education; HS, high school; MLD, mathematical learning disability; TA, typically achieving. Negative values indicate the contrasted group (e.g., girls) had lower scores than the contrast group (e.g., boys), and positive values indicate the opposite. The odds ratio = *e*^estimate^*.

### Variation within the MLD group

Group differences in mean levels of performance (Figure 1) suggest uniform quantitative deficits for children composing the MLD group. Previous studies, however, indicate that even within such groups there are often children who perform relatively well on some quantitative tasks (Denvir and Brown, 1986; Dowker, 2005b; Jordan et al., 2009a). To assess this possibility, we first categorized performance on the ANS task percent correct and on the five tasks (across both times of measurement) shown in Figure 1 as above or below the overall mean (across both groups). Children in the MLD group scored above the mean on an average of 3.5 (range 0–8) of the 11 tasks, as compared to 7.5 (range 2–11) tasks for the TA group [χ^2^(11) = 31.8, *p* = 0.0008]. Only four of the 34 children at risk for MLD scored below average on all 11 tasks. Fifteen of these 34 children had an above average percent correct on the ANS task, and 12 of them scored above average across both times of measurement for one of the five tasks shown in Figure 1; five children scored above average on two tasks and one child on three tasks.

These did not substantially influence group-level mean scores, because different children within the MLD group tended to perform well on different tasks. Three scored above average for both times of measurement for the give-a-number task, four for the numeral recognition and verbal counting tasks, six for the ordinal choice task, and eight for the enumeration task.

## Discussion

The study of children enrolled in Title I preschool is well suited to our goal of identifying early risks of later MLD and LA. Indeed, after a year of preschool, half of the children in our sample had mathematics achievement scores in the same range as those found in school-age children with MLD or LA (Geary et al., 2007; Murphy et al., 2007). It is premature to consider these preschoolers as MLD or LA, but they appear to be at high risk of becoming so, independent of the effects of intelligence, executive functions, and parental education on mathematics achievement. The high proportion of children at risk for MLD or LA allowed us to test the hypothesis that an impaired ANS is the core deficit underlying their poor mathematics achievement (Piazza et al., 2010), and the broader assessment of quantitative competencies allowed us to gauge the importance of the ANS relative to other early competencies that are predictive of later mathematics achievement (Locuniak and Jordan, 2008; Jordan et al., 2009b).

The results provide some support for the hypothesis that an impaired ANS contributes to the mathematics achievement deficits of children at risk for MLD (Piazza et al., 2010; Mazzocco et al., 2011b), but the results are not definitive. The at risk group had, as predicted, higher Weber fractions (i.e., less fidelity) and lower accuracy on the ANS task. Moreover, a 1 SD decrease in ANS task percent correct resulted in a substantial increase in the odds (2.4) of being classified as at risk for MLD after a year of preschool. However, controlling for intelligence, executive control, and preliteracy (letter identification) scores eliminated the group difference for the Weber fraction and attenuated it for percent correct.

On the one hand, these analyses suggest that control of other factors that might affect mathematics learning (intelligence, executive control) is important for testing the ANS hypothesis. On the other hand, Piazza et al. (2010) controlled for intelligence and Mazzocco et al. (2011a) controlled for working memory and still found ANS deficits in school-age children with MLD. One source of the across-study discrepancies is in the characteristics of the samples defined as MLD, or dyscalculic (Piazza et al., 2010). For instance, our sample of at risk children had low average intelligence scores, as is commonly found (Geary et al., 2007), but the children identified as dyscalculic in the Piazza et al. study were of average intelligence. Indeed, our analyses of MLD and TA subgroups matched on intelligence confirmed Piazza et al.’s findings but the results were only significant for ANS task percent correct and not the Weber fraction. No doubt our small sample sizes for these subgroups were a contributing factor. As we recruit additional children into the study, we will be able to obtain a larger sample of preschool children of average intelligence and effortful control and very low mathematics achievement scores and will then be able to provide a more sensitive replication attempt of the Piazza et al. (2010) and Mazzocco et al. (2011a) findings.

Regardless, simultaneous estimation of group differences on the number knowledge composite and ordinal choice tasks eliminated the significance of ANS task percent correct in predicting the odds of MLD status. Thus, for children who are entering preschool the best predictors of risk for MLD, independent of intelligence, executive control, preliteracy scores, and parental education, is poor knowledge of Arabic numerals, number words, and their cardinal values. These children not only began preschool behind their TA peers on these tasks, they fell further behind as the year progressed. These findings do not mean poor ANS fidelity did not contribute to these children’s low mathematics achievement. We suggest that any such deficit may largely operate through ease of learning the relation between Arabic numerals, number words and the magnitudes they represent. This is consistent with Rousselle and Noël’s (2007) hypothesis that mapping symbols onto magnitude representations contributes to the deficits of children with MLD, but further suggests that the fidelity of ANS representations themselves may influence the mapping process.

An unexpected finding was that the children at risk for MLD were unable, even at the end of a year of preschool, to discriminate more and less on the ordinal choice task. Performance on this task is not dependent on an understanding of numerals or number words and may be dependent on properties of the ANS that are not captured by the discrete discrimination task (Gallistel and Gelman, 1992; Gallistel, 2007). Gallistel and Gelman (2005) argued the most important aspect of the ANS is that the generated magnitude representations embody cardinality and ordinality information. However, it will require a larger sample of preschoolers to fully explore whether or not these at risk children’s deficit on this task is related to poor ANS acuity or other properties of this system.

Finally, our results confirm previous studies that have shown that even within MLD/LA groups many children will show normal or better performance in some quantitative areas (Denvir and Brown, 1986; Geary, 1990; Geary et al., 1991) and more generally that the development of quantitative competencies is uneven (Dowker, 2005b; Jordan et al., 2009a). Previous studies of MLD/LA school age children suggest that those with at least some intact quantitative competencies show larger across-grade achievement gains than their peers with deficits in multiple areas (Geary et al., 1991). These results suggest that the children in our at risk group who showed multiple islets of normal performance on the quantitative tasks may not in fact be MLD in the long-term. Follow-up of these children will determine if this is in fact that case. Either way, our results and related ones indicate that many MLD/LA children will have some quantitative strengths that may be potential building blocks for remedial interventions.

### Conclusion

Preschoolers with a strong intuitive sense of quantity, as measured by their ability to quickly determine which of two collections of objects has more (ANS task), score higher on mathematics achievement tests than other children, controlling for intelligence, effortful control, and preliteracy knowledge. Preschoolers at high risk for a learning disability in mathematics have a poor intuitive sense of quantity, but their poor understanding of more and less, and slow learning of Arabic numerals, number words, and their meanings may constitute a stronger long-term risk.

## Conflict of Interest Statement

The authors declare that the research was conducted in the absence of any commercial or financial relationships that could be construed as a potential conflict of interest.
